# Effects of hydroxyproline supplementation in low fish meal diets on collagen synthesis, myofiber development and muscular texture of juvenile Pacific white shrimp (*Litopenaeus vannamei*)

**DOI:** 10.1016/j.aninu.2024.01.013

**Published:** 2024-03-28

**Authors:** Menglin Shi, Haoming Li, Tianyu Chen, Bocheng Huang, Xiaoyue Li, Xiaohui Dong, Shuyan Chi, Qihui Yang, Hongyu Liu, Junming Deng, Beiping Tan, Shuang Zhang, Shiwei Xie

**Affiliations:** a Laboratory of Aquatic Nutrition and Feed, College of Fisheries, Guangdong Ocean University, Zhanjiang 524088, China; b Aquatic Animals Precision Nutrition and High-Efficiency Feed Engineering Research Centre of Guangdong Province, Zhanjiang 524088, China; c Key Laboratory of Aquatic, Livestock and Poultry Feed Science and Technology in South China, Ministry of Agriculture, Zhanjiang 524088, China; d Guangdong Provincial Key Lab of Aquatic Animals Disease Control and Healthy Culture, Zhanjiang 524088, China

**Keywords:** Hydroxyproline, Collagen, Muscle quality, *Litopenaeus vannamei*

## Abstract

This experiment aimed to evaluate the impact of dietary hydroxyproline (Hyp) supplementation on the muscle quality of juvenile Pacific white shrimp (*Litopenaeus vannamei*) fed a low fishmeal diet. Six formulated diets included one high fishmeal (HF; 25% fishmeal content) and five low fishmeal diets (10% fishmeal content) with 0%, 0.2%, 0.4%, 0.6% and 0.8% Hyp (LF0, LF2, LF4, LF6 and LF8, respectively). Each diet was assigned to four replicates, and 40 shrimp (0.32 ± 0.00 g) per replicate were fed four times a day for 8 weeks. Dietary Hyp supplementation had little effects on growth performance, but increased the contents of Hyp, prolyl 4-hydroxylases (P4Hs), and collagen. The meat yield, springiness, hardness, chewiness, and cohesiveness of muscle were the highest in the LF4 group among the low fishmeal groups (*P* < 0.05). Cooking loss and freezing loss of muscle were the lowest in the LF4 group (*P* < 0.05). Dietary supplementation with 0.4% Hyp increased the myofiber density and decreased the myofiber diameter of muscle (*P* < 0.05). Supplementation of Hyp in the diet up-regulated the mRNA expression of *smyhc5*, *smyhc15*, *col1a1*, *col1a2*, *igf-1f*, *tgf-β* and *tor* and down-regulated the mRNA expression of *smyhc1*, *smyhc2*, *smyhc6a* (*P* < 0.05). Supplementation of Hyp in the diet up-regulated the protein expression of P-4E-BP1, P-AKT, AKT and P-AKT/AKT (*P* < 0.05). These results suggested that the addition of 0.4% Hyp to low fishmeal diets improved the muscle quality of *L. vannamei*.

## 1 Introduction

Shrimp has delicious taste, high protein and micro-nutrient content, and is regarded as a kind of health food by people (Tapsell et al., 2016). Among the various shrimp species, Pacific white shrimp (*Litopenaeus vannamei*) is the most popular due to its quick growth, heat tolerance, and high yield (Chen et al., 2023; Zhang et al., 2018). However, themuscle quality of *L. vannamei* has been observed to deteriorate during mass farming, which can negatively affect consumer satisfaction. It is critical to improve the muscle quality of shrimp in order to satisfy customer demand.

The quality of muscle is determined by a combination of physical properties, such as nutritional composition, special flavor, and other characteristics. The sensory evaluations of food, such as texture and flavor, are influenced by a wide range of biological factors, such as muscle protein content, lipid content, and amino acid composition (Chen et al., 2021). The nutritional content of the feed fed to shrimp affects the flesh and muscle quality of shrimp. Despite fishmeal being an important nutrient in shrimp diets, it is in limited supply, and so needs to be replaced by other protein sources (Shi et al., 2023a, Shi et al., 2023b). Bacterial proteins and plant proteins are new protein sources for aquatic animals with great potential to replace fishmeal (Zheng et al., 2022). The most prevalent protein sources are *Clostridium autoethanogenum* protein (CAP) and soy protein concentrate (SPC). However, the nutrition of CAP and SPC is not as comprehensive as in fishmeal, which results in adverse effects on aquatic animals (Shi et al., 2023a, Shi et al., 2023b). Fish meal has the characteristics of high protein content, good palatability, balanced amino acids, and easy digestion and absorption. It has always been regarded as the most nutritious and highly digestible protein source for aquatic feed. Soy protein concentrate and most plant proteins have the disadvantage of poor palatability and low lipid content. Previous studies have shown that substituting a large amount of fish meal with SCP can have detrimental effects on growth performance (Paripatananont et al., 2001). Similarly, CAP has the disadvantage of low lipid content, and previous studies have shown that excessive substitution of fish meal with CAP can inhibit shrimp growth (Yao et al., 2022). The substitution of CAP and SPC for dietary fish meal decreases the quality of shrimp flesh (Yao et al., 2022; Zhang et al., 2019). Previous studies indicated that SPC is rich in anti-nutritional factors that can cause damage to muscle cells, resulting in muscle texture damage (Fan et al., 2022). *C. autoethanogenum* protein reduces lipid contents in muscle and also affects the synthesis of collagen (Yang et al., 2022a,b). Researchers have shown that feed additives can improve shrimp muscle quality, and that dietary supplementation with β-hydroxy-β-methylbutyrate can improve muscle hardness, water holding capacity, and collagen content (Mu et al., 2023).

Hydroxyproline (Hyp) is a semi-essential amino acid for aquatic animals, andis abundant in fishmeal and animal protein but deficient in plant proteins (Wu et al., 2011). Hyp is an amino acid specific to collagen, indispensable for collagen synthesis, and can be found at the Y position within the collagen helix region (Liu et al., 2014). Collagen plays an important role in muscle integrity and toughness, and research has suggested that adding Hyp to feed can improve the quality of muscle in large yellow croaker (*Larimichthys crocea*) (Wei et al., 2018). The addition of 0.74% Hyp to the feed significantly improved the protein synthesis in triploid crucian carp muscle (*Carassius auratus Triploid*) (Cao et al., 2022). The addition of Hyp in the feed significantly improved the collagen content and muscle quality of Chinese perch (*Siniperca chuatsi*) (Feng et al., 2022). Current research on the use of Hyp in improving muscle quality in aquatic animals has mainly focused on fish, with little research on the *L. vannamei* muscle quality. Therefore, this study was designed to inquire into the effect of Hyp on the growth parameters, muscle quality, and myofiber properties of *L. vannamei*.

## 2 Materials and methods

### 2.1 Animal ethics statement

The animal protocol used in the present study was approved by the ethics review board of Guangdong Ocean University (approval ID: GDOU-IACUC-2022-A0502; approval date: 2 May 2022), and all experimental procedures concerning animals were performed in accordance with the National Center for the Replacement Refinement & Reduction of Animals in Research (The ARRIVE guidelines 2.0) and relevant regulations.

### 2.2 Diet preparation

In this experiment, six diets were formulated as follows: one containing 25% fishmeal (named HF meaning high fish meal) and other five diets containing 10% fishmeal and supplemented with 0%, 0.2% 0.4% 0.6% and 0.8% Hyp (named LF0, LF2, LF4, LF6, and LF8, respectively). Hyp was supplied by Aladdin Reagent Co., Ltd (Shanghai, China). To attain the same nutritional composition of HF diet, fish oil, soybean oil, and methionine were added to the five low fishmeal diets. The raw materials were crushed, sieved and weighed using an 80-mesh sieve, and the raw materials were mixed by the step expansion method and finally homogeneously mixed using a V-shaped mixer (M-256, South China University of Technology, Guangdong, China). Each group of well-mixed ingredients was extruded into 1.0-mm and 1.5-mm diameter dies in a twin-screw extruder (School of Chemical Engineering, South China University of Technology, Guangdong, China). The diets were matured at 85 °C for 30 min and stored in a −20 °C refrigerator. Table S1 shows the diet formulation and proximate compositional analysis.

### 2.3 Shrimp and experimental conditions

*L. vannamei* was purchased from Haixing Agriculture Co., Ltd (Zhanjiang, China) and transported to the base of Guangdong Ocean University in Donghai island, Zhanjiang, China. The shrimp were fed with a commercial feed for one week before the experiment to adjust to the experimental environment. After 24 h of fasting, 960 juvenile shrimps (0.32 ± 0.00 g) were selected, weighed and placed in six groups of tanks with four replicates of 40 shrimps each. The experiments were conducted in an indoor culture environment with four feedings per day at 07:20, 11:30, 16:30 and 21:30, and feed intake was recorded 40 min after each feeding. Daily feeding amount was adjusted based on experience, daily feeding intake and mortality rate (diet size: 1.0 mm diameter feed for the first 21 d; diet size: 1.5 mm diameter feed for the last 30 d). In the case of bad weather such as typhoons, rainstorms and thunderstorms, feedings may have be reduced or even suspended. Approximately 60% of the water was changed daily to maintain water quality. The water conditions were as follows: temperature held in the range of 25 to 28 °C, dissolved oxygen >7 mg/L, and nitrates <0.05 mg/L, which were tested by a multi-parameter water quality detector (PTF-001B, WBD Biotechnology Co., Ltd., China).

### 2.4 Sample collection and chemical analyses

The shrimp were starved for 24 h after a 51-d feeding period before anesthesis, weighing, and counting for each tank. Before collecting samples, the shrimp was anesthetized in an ice water bath (0 to 4 °C). Four shrimp were selected from each tank to measure body composition, Hyp and proline. Three shrimp were weighed to calculate meat rates; and three shrimp were used to assess freezing loss. Muscle tissue (2 mm × 2 mm × 2 mm) from each group was collected for analysis by electron microscopy and H&E staining. In addition, three shrimp were selected for cooking loss and texture analysis. Finally, the hepatopancreas and muscle of four shrimp from each tank were collected, later stored with RNA (Ambion, USA), placed in a refrigerator at 4 °C for 11 h and rapidly frozen in an ultra-low temperature refrigerator at −80 °C for subsequent gene expression analysis.

The AOAC (2005) methods were used to conduct the nutritional composition analysis on crude protein, crude lipid, amino acids, and moisture content of muscle and diets. Moisture was removed from sample by heating in an oven at 105 °C until at constant weight. The crude protein contents were determined by the Dumas nitrogen method with a Primacs100 analyzer (Skalar, Dutch). The crude lipids were measured using an XT15 extractor. The amino acid composition of the diets was measured by Evonik Investment Co., Ltd (Bejing, China) as shown in Table S2.

The Hyp content was measured in the muscle, hemolymph, and hepatopancreas using a commercial kit (A030-2-1, Nanjing Jiancheng Bioengineering Institute, China). First, the sample was hydrolyzed in boiling water for 20 min. After cooling, the pH value of the solution was adjusted to about 6.0 to 6.8 by adding pH solutions A and B. It was thenmixed with double distilled water and activated charcoal. Next, the solution was centrifuged at 875 × *g* for 10 min and then the supernatant was taken and tested with an UV–VIS spectrophotometer (UV-5800PC, Shanghai Yuanxi Instrument Co. Ltd., China) at a wavelength of 550 nm. Prolyl 4-hydroxylases (P4Hs) were quantified in muscle, hemolymph, and hepatopancreas using an ELISA kit (Enzyme-linked Biotechnology Co. Ltd., Shanghai, China). The determination of collagen content was achieved through the application of method 990.26 (AOAC, 2005).

### 2.5 Real-time quantitative PCR (RT-qPCR)

Extraction of RNA and RT-qPCR were performed according to the method of Zhao et al. (2022). Total RNA of the samples was extracted using Trizol Reagent (Transgen Biotech, China). Purity was determined by 1% agarose gel electrophoresis. The cDNA was reverse transcribed from mRNA by the QuantiTect Reverse Transcription Kit (Accurate Biotechnology, China). The primers were synthesized (Sangon Biotech Co., Ltd., Guangdong, China) and the sequences are listed in Table S3. Real-time PCR for the target genes was performed using a SYBR Green Premix Pro Taq HS qPCR Kit II (Accurate Biotechnology (Hunan) Co., Ltd, Changsha, China). Quantitative analysis was performed on LightCycler 480 (Roche Applied Science).

### 2.6 Flesh texture

The shrimp meat was cooked in boiling water for 3 min, then removed from the water and wiped dry. It was allowed to equilibrate at room temperature for 1 h before measurement. The muscle texture, including hardness, springiness, chewiness and cohesiveness, was determined using a texture analyzer (TA.XT PlusC, SMS, USA). The measurement was taken as follows. On the second section of the shrimp muscle, two compressions were performed on the section using a P/0.5 probe at a test speed of 1 mm/s. A target strain mode of 40% strain is applied for 5 s.

### 2.7 Water-holding capacity

A 3-g shrimp muscle was used to measure cooking loss and any surface moisture was removed before weighing. After boiling the muscle in water for 3 min, it was removed from the water and allowed to cool to room temperature. Then, any surface moisture was meticulously eliminated before conducting a second weighing.

Steaming loss (%) = 100 × (*W*_*t*_ − *W*_*i*_)/*W*_*t*_,

where *W*_*t*_ is the weight of the shrimp before cooking (g), *W*_*i*_ is the weight of cooked shrimp (g).

To determine freezing loss, a shrimp muscle sample weighing approximately 3 g was selected and its surface moisture was dried before weighing. Afterward, the sample was chilled at −20 °C for 24 h and then allowed to thaw at room temperature until fully defrosted. Once it was thawed, the surface was carefully dried and it was reweighed.

Freezing loss (%) = 100 × (*W*_*a*_ − *W*_*b*_)/*W*_*a*_,

where *W*_*a*_ is the weight of the shrimp before freezing (g), *W*_*b*_ is the weight of frozen shrimp (g).

### 2.8 Muscle paraffin and electron micrographs analysis

Muscle from 2 shrimp per tank was fixed in Bouin's fixative for 24 h, followed by dehydration in 70% ethanol. Subsequently, the muscle was embedded in paraffin after undergoing dehydration using different levels of ethanol. H&E staining was performed, and observation was made using a microscope (Ni-U, Nikon, Japan). Additionally, two muscle samples measuring 2 mm × 2 mm × 2 mm were taken for transmission electron microscopy (TEM) and analyzed (Wuhan Seville Biotechnology Co., Ltd, Hubei, China). Myofiber width measurements were conducted using ImageJ software, and a total of 10 random measurements were taken within the same section.

### 2.9 Western blot analyses

Thirty milligrams of hepatopancreatic tissue were extracted and subsequently treated with PBS, cell lysate, protease inhibitor, and phosphorylase inhibitor. Finally, phenylmethylsulfonyl fluoride (PMSF) was added to facilitate disruption. Muscle protein concentration was measured using a BCA protein assay kit (Beyotime, Shanghai), and the concentration was adjusted to 4 mg/mL using a PBS loading buffer. After it was denatured for 10 min in boiling water, a total of 36 mg of protein was added to each well, and sodium dodecyl sulfate-polyacrylamide gel electrophoresis (SDS-PAGE) was used for protein separation. After electrophoresis, the membrane was transferred to the quick blocking solution and blocked for 25 min at room temperature. After detection using an ECL chemiluminescence kit (Billerica, MA, United States), the protein bands were imaged using a fully automated chemiluminescence image analysis system. The bands were quantified using Image J (version 1.43, National Institutes of Health). In this study, the following antibodies were used: antibodies against phosphor-4E-BP1(Thr^37/46^; rabbit no. 9459; Cell Signaling Technology), 4E-BP1(rabbit no. 9452; Cell Signaling Technology), phosphor-AKT (Ser^473^; rabbit no. 9271S; Cell Signaling Technology), AKT (rabbit no. 9272S; Cell Signaling Technology), a-Tubulin (rabbit no. AF7010; Affinity).

### 2.10 Calculations and statistical analysis

The parameters were calculated as follows:

Weight gain rate (WGR, %) = 100 × (*W*_*1*_ − *W*_*0*_)/*W*_*1*_,

Specific growth rate (SGR, %/day) = 100 × (ln *W*_*1*_ − ln *W*_*0*_)/*t*,

Survival rate (SR, %) = 100 × *N*_*1*_/*N*_*0*_;

Feed conversion rate (FCR) = feed consumed/(*W*_*1*_ − *W*_*0*_),

Feed intake (FI) = feed consumed/survival number,

Meat yield (%) = 100 × (weight of the abdominal and tail muscle/final weight),

where *t* is the experimental duration in days; *W*_*0*_ is the initial body weight (g); *W*_*1*_ is the final body weight (g); *N*_*0*_ is the initial number of shrimp; and *N*_*1*_ is the final number of shrimp.

The results were presented as the means ± SEM. All data were tested for normality (Kolmogorov–Smirnov test) and homogeneity (Levene's test). The one-way analysis of variance (ANOVA) was conducted to determine significantly differences among individual data and the Tukey's was conducted to identify significant differences among treatments. Following the ANOVA, the dates were tested in SPSS 21.0 to (SPSS, Chicago, IL, USA). A probability value of *P* < 0.05 was deemed to be statistically significant.

## 3 Results

### 3.1 Growth performance and composition of muscle

The growth performance and muscle quality results are shown in Table 1. Final body weight (FBW), WGR and SGR of *L. vannamei* fed diet LF8 were lower than those fed diet LF4 (*P* < 0.05). The FCR of *L. vannamei* fed diet LF4 was lower than those fed diets HF, LF0 and LF8 (*P* < 0.05). There is no effect on SR and FI among all the trial groups (*P* > 0.05). In addition, the meat yield of HF group was the highest, which was higher than other groups except for the LF4 group (*P* < 0.05). As shown in Table S4, the results in moisture, crude protein, and crude lipid of the muscle were similar (*P* > 0.05).

**Table 1:** Growth performance of *L. vannamei* fed different diets.

Index	Diet^1^					
	HF	LF0	LF2	LF4	LF6	LF8
FBW, g	8.66 ± 0.245^ab^	8.67 ± 0.277^ab^	8.89 ± 0.153^ab^	9.29 ± 0.115^b^	8.57 ± 0.108^ab^	8.36 ± 0.171^a^
WGR, %	2604.74 ± 76.126^ab^	2610.34 ± 87.146^ab^	2679.12 ± 48.219^ab^	2803.86 ± 35.255^b^	2579.53 ± 33.462^ab^	2509.97 ± 53.651^a^
SGR, %/day	6.34 ± 0.051^ab^	6.34 ± 0.062^ab^	6.4 ± 0.034^ab^	6.48 ± 0.022^b^	6.32 ± 0.023^ab^	6.27 ± 0.037^a^
SR, %	93.67 ± 0.667	95.33 ± 2.667	96.67 ± 1.667	96.00 ± 1.000	94.33 ± 3.480	93.67 ± 2.333
FCR	1.40 ± 0.028^c^	1.35 ± 0.054^bc^	1.26 ± 0.028^ab^	1.22 ± 0.040^a^	1.29 ± 0.041^abc^	1.40 ± 0.041^c^
FI, g/shrimp	11.63 ± 0.310	11.24 ± 0.089	10.78 ± 0.167	10.91 ± 0.238	10.66 ± 0.250	11.21 ± 0.340
Meat yield, %	46.40 ± 0.867^d^	42.99 ± 0.432^b^	40.89 ± 0.208^a^	46.22 ± 0.298^cd^	44.50 ± 0.164^bc^	40.75 ± 0.223^a^

FBW = final body weight; WGR = weight gain rate; SGR = specific growth rate; SR = survival rate; FCR = feed conversion rate; FI = feed intake.

Data represent mean ± SEM of three replicates (*n* = 3). Values in the same row with different superscripts are significantly different (*P* < 0.05) based on Tukey's multiple-test.

1 HF = high fishmeal (25% fishmeal content); LF0 = low fishmeal (10% fishmeal content); LF2 = LF0 + 0.2 g/kg hydroxyproline; LF4 = LF0 + 0.4 g/kg hydroxyproline; LF6 = LF0 + 0.6 g/kg hydroxyproline; LF8 = LF0 + 0.8 g/kg hydroxyproline.

### 3.2 The contents of Hyp and P4Hs

**Fig. 1:**
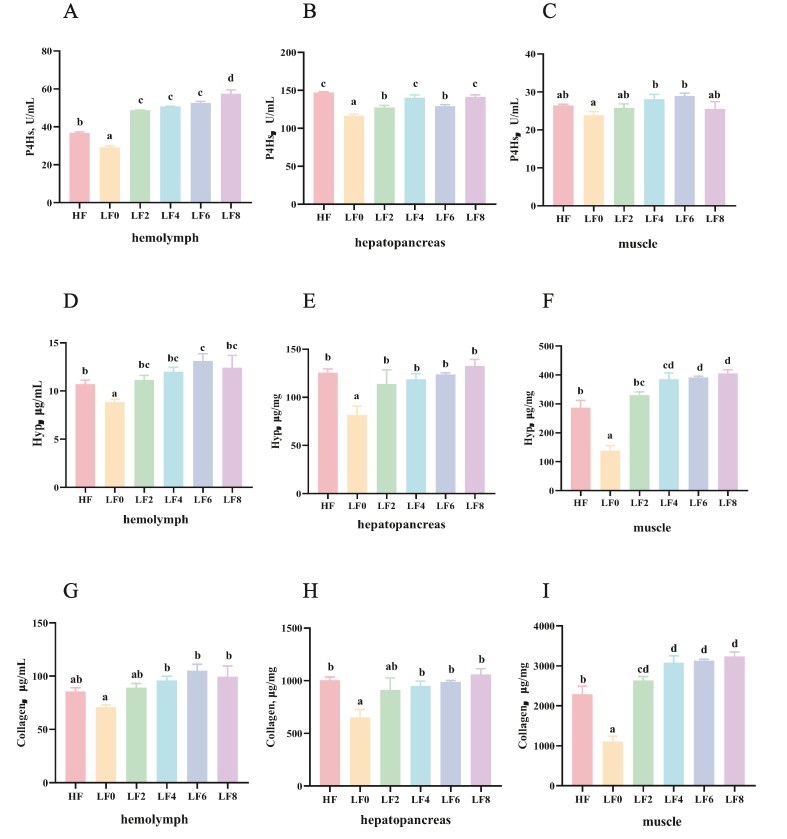
Effect of hydroxyproline (Hyp) addition to low fishmeal diets on the content of prolyl 4-hydroxylases (P4Hs), Hyp and collagen in hemolymph, hepatopancreas and muscle of *L. vannamei*. (A to C) The P4Hs content in the hemolymph, hepatopancreas and muscle. (D to F) The Hyp content in the hemolymph, hepatopancreas and muscle. (G to I) The collagen content in the hemolymph, hepatopancreas and muscle. HF = high fishmeal; LF0 = low fishmeal; LF2 = LF0 + 0.2 g/kg Hyp; LF4 = LF0 + 0.4 g/kg Hyp; LF6 = LF0 + 0.6 g/kg Hyp; LF8 = LF0 + 0.8 g/kg Hyp. Bars with different superscripts are significantly different (*P* < 0.05).

The contents of P4Hs in hemolymph, hepatopancreas, and muscle are shown in Fig. 1A to C. Hyp supplementation increased the content of P4Hs in hemolymph and hepatopancreas (*P* < 0.05). In muscle, the content of P4Hs was lower in the LF0 group than in the LF4 and LF6 groups (*P* < 0.05).

Figure 1D to F shows the content of Hyp in hemolymph, hepatopancreas, and muscle. The Hyp content in hemolymph, hepatopancreas, and muscle was lower in the LF0 group than in the other experiment groups (*P* < 0.05). However, the hemolymph Hyp content of *L. vannamei* which fed the LF6 diet getting the maximum value was higher than the group fed the LF0 diet (*P* < 0.05). The content of Hyp in HF group was lower than LF4, LF6 and LF8 groups (*P* < 0.05). As shown in Fig. 1G to I, collagen contents in the hemolymph, hepatopancreas, and muscle of *L. vannamei* were significantly lower in the LF0 group comparing with other experimental groups (*P* < 0.05). The collagen content in the muscle of *L. vannamei* was lower in the LF0 group comparing with other experimental groups (*P* < 0.05), and at the same time, the collagen content in the LF4, LF6, and LF8 groups was significantly higher than the HF group (*P* < 0.05).

### 3.3 Collagen synthesis and myofibers development gene expression

**Fig. 2:**
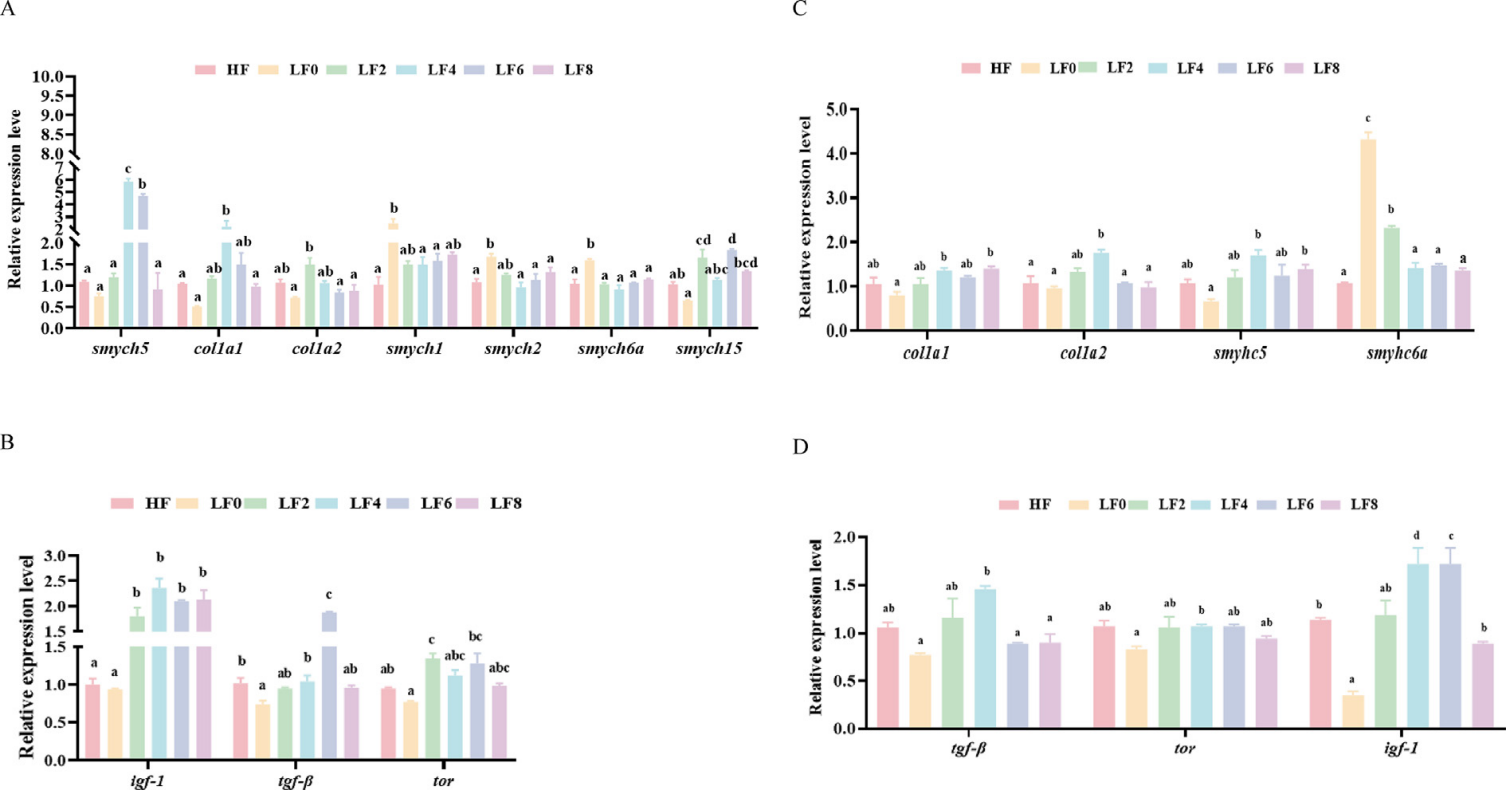
Effect of hydroxyproline (Hyp) addition to low fishmeal diets on the expression of muscle quality-related genes in the muscle and hepatopancreas of *L. vannamei*. (A to B) The expression of muscle quality-related genes in the muscle. (C to D) The expression of muscle quality-related genes in the hepatopancreas. *tgf-β* = transforming growth factor-beta receptor-associated protein 1-like; *igf-1* = insulin-like growth factor-binding protein-related protein 1; *tor* = target of rapamycin complex 2 subunit mapkap1 -like; *smych 1*, *smych 2*, *smych 6a*, *smych 5*, *smych 15* = myosin heavy chain gene family; *col1a2* = collagen alpha-2(I) chain-like; *col1a1* = collagen alpha-1(I) chain-like. HF = high fishmeal; LF0 = low fishmeal; LF2 = LF0 + 0.2 g/kg Hyp; LF4 = LF0 + 0.4 g/kg Hyp; LF6 = LF0 + 0.6 g/kg Hyp; LF8 = LF0 + 0.8 g/kg Hyp. Bars with different superscripts are significantly different (*P* < 0.05).

Muscle quality-related gene expression is shown in Fig. 2A and B. The expression of *smyhc5*, and *col1a1* in shrimp fed the diets supplemented with Hyp showed an increase and then decreased in the groups with high doses of Hyp (*P* < 0.05). Compared with the LF0 group, Hyp down-regulated the mRNA expression of *smyhc1*, *smyhc2*, and *smyhc6a* but up-regulated the mRNA expression of *igf-1*, *tgf-β*, and *tor*. The gene expression of *col1a2* was the highest in LF2 group. Beyond that, the mRNA expression of *smyhc15* was the highest in *L. vannamei* fed diet LF6, compared with other groups (*P* < 0.05).

Muscle quality-related gene expression in hepatopancreas is shown in Fig. 2C and D. The expression of *col1a2*, *smyhc5*, and *igf-1* in the LF4 group were higher (*P* < 0.05) than other groups. Compared with the LF0 group, dietary supplementation with Hyp not only down-regulated the mRNA expression of *smyhc6a* but also up-regulated the mRNA expression of *igf-1* and *col1a1*(*P* < 0.05).

### 3.4 Flesh texture characteristics, water-holding capacity

As shown in Table 2, Hardness, chewiness and cohesiveness in LF0 group were lower than those in HF group (*P* < 0.05), and hardness, springiness, chewiness and cohesiveness of the LF4 group were higher than those in the LF0 group (*P* < 0.05). In addition, flesh cooking loss of the LF0 group was higher than that of other groups (*P* < 0.05), and the freezing loss of the LF4 group was lower than that of the LF0 group (*P* < 0.05). However, there are no significant effects on flesh hardness, chewiness, chewiness, cohesiveness, cooking loss, and freezing loss in the LF4 group, compared with the HF group (*P* > 0.05).

**Table 2:** Effect of hydroxyproline (Hyp) addition to low fishmeal diets on muscle texture, water-holding capacity of *L. vannamei*.

Index	Diet^1^					
	HF	LF0	LF2	LF4	LF6	LF8
Hardness, N	1044.10 ± 31.480^c^	791.16 ± 15.368^ab^	957.94 ± 13.476^bc^	1000.43 ± 37.722^c^	886.67 ± 52.341^abc^	768.93 ± 35.092^a^
Springiness, mm	0.62 ± 0.005^bc^	0.54 ± 0.017^a^	0.60 ± 0.014^ab^	0.66 ± 0.010^c^	0.65 ± 0.013^bc^	0.63 ± 0.015^bc^
Chewiness, mJ	370.44 ± 30.884^c^	244.31 ± 13.559^ab^	227.44 ± 27.130^a^	374.08 ± 28.442^c^	349.98 ± 20.908^bc^	352.21 ± 17.018^bc^
Cohesiveness, N	0.56 ± 0.012^c^	0.46 ± 0.013^a^	0.5 ± 0.014^ab^	0.56 ± 0.009^c^	0.53 ± 0.008^bc^	0.53 ± 0.011^bc^
Cooking loss, %	8.46 ± 1.007^a^	11.66 ± 0.723^b^	7.92 ± 0.429^a^	7.49 ± 0.830^a^	8.31 ± 0.283^a^	8.56 ± 0.429^a^
Freezing loss, %	0.85 ± 0.010^ab^	1.27 ± 0.006^b^	1.13 ± 0.02^b^	0.49 ± 0.080^a^	0.94 ± 0.152^ab^	1.22 ± 0.136^b^

Data represent means ± SEM of three replicates (*n* = 3). Values in the same row with different superscripts are significantly different (*P* < 0.05) based on Tukey's multiple-test.

1 HF = high fishmeal; LF0 = low fishmeal; LF2 = LF0 + 0.2 g/kg Hyp; LF4 = LF0 + 0.4 g/kg Hyp; LF6 = LF0 + 0.6 g/kg Hyp; LF8 = LF0 + 0.8 g/kg Hyp.

### 3.5 Myofibrillar morphology

**Fig. 3:**
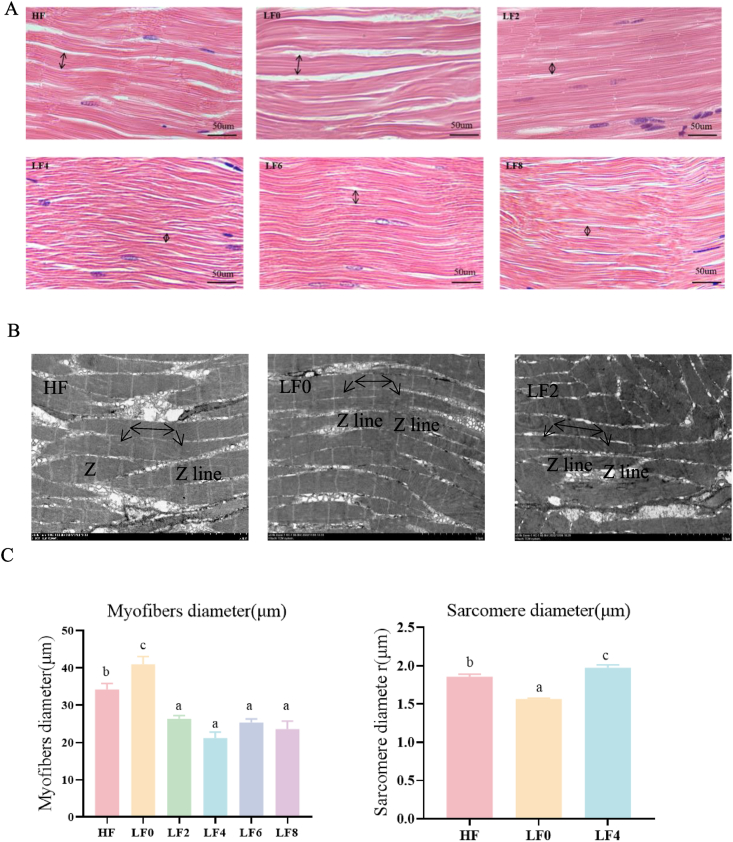
Effect of hydroxyproline (Hyp) addition to low fishmeal diets on the morphology of muscle fibers of *L. vannamei*. (A) Myofiber microstructure of longitudinal sections. (B) Transmission electron microscope of longitudinal sections. (C) Myofibers diameter and sarcomere diameter. HF = high fishmeal; LF0 = low fishmeal; LF2 = LF0 + 0.2 g/kg Hyp; LF4 = LF0 + 0.4 g/kg Hyp; LF6 = LF0 + 0.6 g/kg Hyp; LF8 = LF0 + 0.8 g/kg Hyp. Bars with different superscripts are significantly different (*P* < 0.05).

As shown in Fig. 3A, the myofibers of the LF2, LF4, LF6 and LF8 groups were more densely packed and the diameter of the myofibers in the Hyp supplemented groups was smaller compared to the HF and LF0 groups. As shown in Fig. 3C, myofiber diameters were smaller in the LF2, LF4, LF6 and LF8 groups than in the HF and LF0 groups (*P* < 0.05). The myofiber diameter of the HF group was smaller than that of the LF0 group, and sarcomere diameter was shorter in the HF and LF0 groups than LF4 groups (*P* < 0.05). TEM of longitudinal myogenic fiber sections of myogenic fibers showed obvious alternating lines (A bands), bright areas (I bands) and Z lines. The sarcomere diameter of the LF4 and HF groups was longer than that of the LF0 group (*P* < 0.05).

### 3.6 Western blot analysis of protein deposition related protein expression

**Fig. 4:**
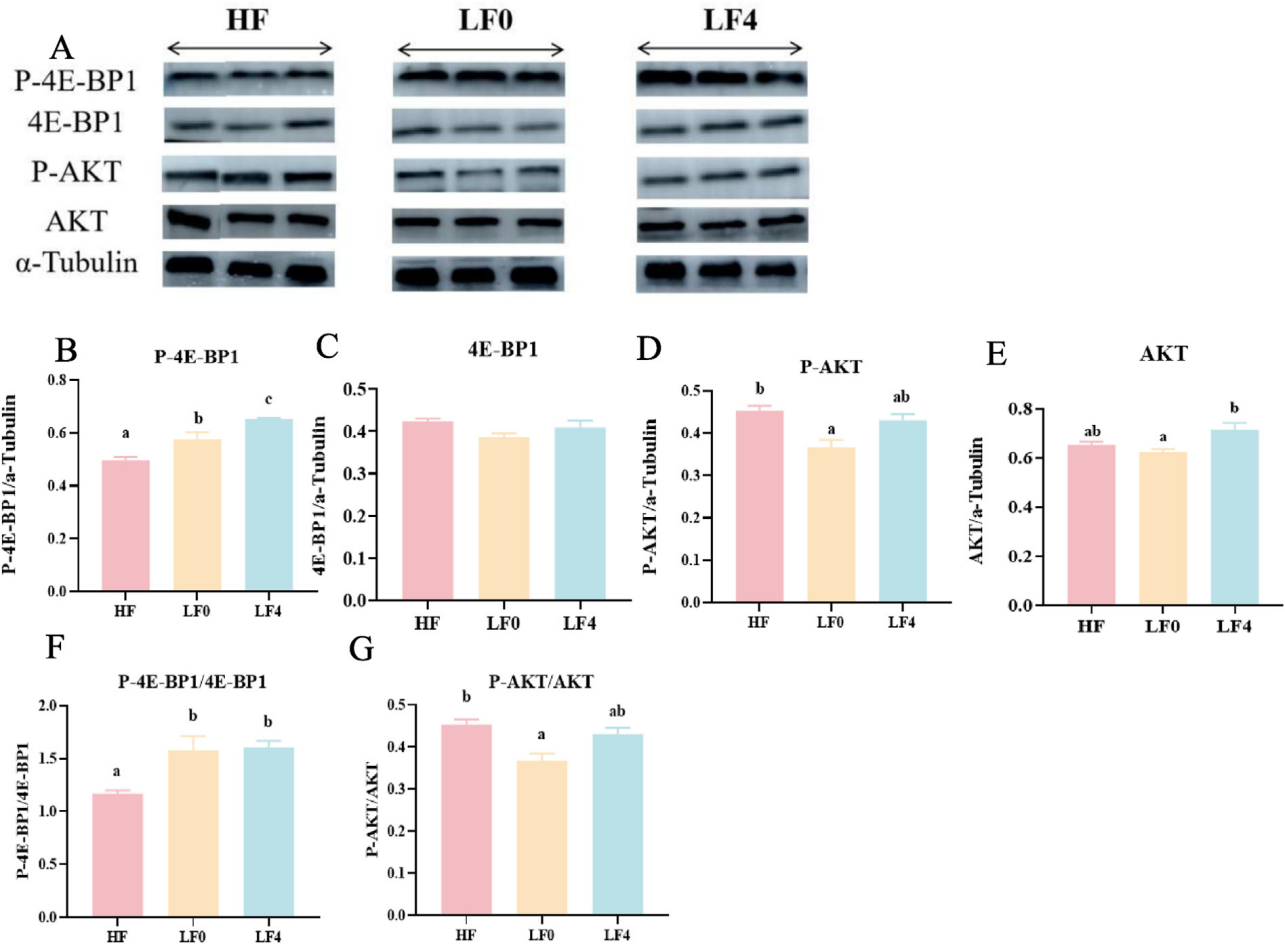
Effect of hydroxyproline (Hyp) addition to low fishmeal diets on the protein deposition of *L. vannamei*. (A) The Western blot analysis of P-4E-BP1,4E-BP1, P-AKT, AKT and α-Tubulin in muscle. (B to E) The relative quantification of protein levels of P-4E-BP1, 4E-BP1, P-AKT and AKT normalized to the α-Tubulin. (F to G) Phosphorylation levels of 4E-BP1 and AKT. P-4E-BP1 = phosphor-4E-binding protein; 4E-BP1 = 4E-binding protein; P-AKT = phosphor-protein kinase B; AKT = protein kinase B. HF = high fishmeal; LF0 = low fishmeal; LF2 = LF0 + 0.2 g/kg Hyp; LF4 = LF0 + 0.4 g/kg Hyp; LF6 = LF0 + 0.6 g/kg Hyp; LF8 = LF0 + 0.8 g/kg Hyp. Bars with different superscripts are significantly different (*P* < 0.05).

As shown in Fig. 4, compared with LF0 diets, dietary supplementation of 0.4% Hyp upregulated the protein expression of P-4E-BP1, P-AKT, AKT and P-AKT/AKT in muscle (*P* < 0.05). In comparison to HF diets, LF0 diets and dietary supplementation of 0.4% Hyp up-regulated the protein expression of P-4E-BP1/4E-BP1 (*P* < 0.05). Among the groups, the protein expression of 4E-BP1 in muscle was similar (*P* > 0.05).

## 4 Discussion

As Hyp content increased in the diet, a trend of increasing and then decreasing was observed in FBW, WGR and SGR, which indicated that the moderate addition of Hyp was beneficial to the growth of *L. vannamei*. Hyp has a significant impact on the structure and physical properties of collagen, so even small changes can have a major impact on individuals (Aksnes et al., 2008; Brinckmann et al., 2005). Excessive hydroxyproline may affect the synthesis of collagen, which is detrimental to the growth of shrimp. Currently, there is insufficient research on this mechanism, and further studies are needed in the future. Consistent with this study, excessive Hyp supplementation in diets negatively affects the growth of Chinese perch (Feng et al., 2022). It has also been demonstrated that supplementing the diets of Yellow River carp (*Cyprinus carpio haematopterus*) with Hyp has no significant effect on the growth performance (Song et al., 2022), with a similar effect observed insalmon (*Salmo salar* L.) (Aksnes et al., 2008). This could be attributed to the fact that different aquatic animals have varying nutritional needs.

It has been found that supplementing feed with Hyp has been shown to improve the muscle quality of turbot (*Scophthalmus maximus* L.) (Liu et al., 2014), Chinese perch (Feng et al., 2022), large yellow croaker (Wei et al., 2018) and triploid crucian carp (Cao et al., 2022). Hyp is crucial for the synthesis of collagen, which ultimately determines the texture of the meat so the amount of Hyp also affects the quality of the muscle. Studies have shown that exogenously ingested Hyp is not further metabolized away, but is immediately absorbed and then conveyed to the tissues (Pinilla-Tenas et al., 2003). This is similar toour results. In this study, dietary supplementation with Hyp significantly increased Hyp levels in hemolymph, muscles, and hepatopancreas. Similar results were previously observed in Chinese perch (Feng et al., 2022) and triploid crucian carp (Cao et al., 2022), where Hyp content in skin, muscle, vertebrae, and hepatopancreas increased with dietary Hyp content. Hyp is an amino acid that plays a key role in maintaining the structural integrity of collagen and is the characteristic amino acid of collagen. In particular, the results of this experiment indicated a significant increase in collagen content in the hemolymph, hepatopancreas, and muscle tissues, as the Hyp content increased. Collagen has intact collagen molecules and is the main component of muscle connective tissue, forming a complete elastic network that has an important impact on muscle hardness (Sikorski et al., 1984). The texture of the muscle is a crucial factor in determining the overall quality of the muscle. In this experiment, muscle hardness, chewiness, elasticity, and cohesiveness increased significantly as the content of Hyp in the diet increased, which may be attributed to an uptick collagen content in the muscles. Consistent with this study, in studies of Atlantic salmon (*S. salar* L.) and grass carp (*Ctenopharyngodon idellus*), higher collagen levels were also found to be associated with greater muscle stiffness (Moreno et al., 2012; Yang et al., 2020). The results mentioned above demonstrated that Hyp supplementation can significantly increase the concentration of synthetic collagen in shrimp, thus improving the muscle quality. Therefore, Hyp supplementation can be considered as a valuable strategy for improving the quality of shrimp muscle.

Collagen content is also positively correlated with muscle water-holding capacity. Muscle water-holding capacity reflects the amount of fluid and soluble substances lost (Brinker and Reiter, 2011). Soluble substances include soluble protein, soluble flavor substances, and heme, and all of them together affect the muscle quality (Luciano et al., 2009). There are two ways to measure the water-holding capacity of muscle: cooking loss and freezing loss. Freezing loss can change the moisture content and distribution of muscles and cooking loss refers to the loss of liquid and soluble substances in muscles during heating (Huff-Lonergan and Lonergan, 2005; Solo-de-Zaldívar et al., 2014). The smaller the values of freezing loss and cooking loss, the stronger water-holding capacity and higher quality of the muscles (Wang et al., 2015). Muscle quality is impacted by the number and shape of connective tissue in the muscle, and a membrane sheath can prevent water from evaporating from the muscle (Purslow, 2005). In this study, the cooking loss and freezing loss of LF4 group were not significantly different from those of HF group, but significantly lower than those of LF0 group. The above results indicated that supplementation with Hyp can increase collagen concentration in low fishmeal diets to improve muscle quality.

In shrimp muscle, type I collagen has been extensively studied and is considered the most well-researched of the five types of collagen, has two α1 chains (*col1a1*) and one α2 chain (*col1a2*) (Mizuta et al., 1994). The two genes *col1a1* and *col1a2* are regulated by the two α1 chains and a α2 chain (Büttner et al., 2004). In this experiment, Hyp supplementation in low fish meal diets up-regulated *col1a1* and *col1a2* expression. The findings of this study are consistent with the results of the triploid crucian carp study, where the mRNA expression of *col1a1* and *col1a2* was up-regulated by the addition of Hyp (Cao et al., 2022). Collagen synthesis involves complex translations and modifications, and P4Hs play a crucial role in collagen synthesis (Myllyharju, 2008) According to this study, it was observed that Hyp supplementation in low fishmeal diets significantly increased the content of P4Hs in hemolymph, hepatopancreas, muscle. This result was consistent with a significant up-regulation of the relative expression of *col1a1* and *col1a2*. The *tgf-β* gene plays a crucial role in regulating type I collagen in mammals, and research on mice has demonstrated that *tgf-β* can significantly enhance the expression of *col1a1* (Liao et al., 2006). *Tgf-β* and *colla1* were up-regulated simultaneously in grass carp (Yu et al., 2019). The study revealed that the expression of the *tgf-β* was significantly up-regulated with the addition of Hyp. In fish, it can promote the proliferation and differentiation of myoblasts (Fuentes et al., 2013). Protein accumulation is a crucial factor in determining the development of shrimp muscles, which in turn is determined by protein synthesis (Dumas et al., 2007). *Tor* signaling pathway influences protein synthesis, and *igf-1* is an essential regulator of the upstream *tor* signaling system, therefore both *tor* and *igf-1* can accelerate protein synthesis (Irm et al., 2020; Picha et al., 2008). Activation of the *tor* signaling pathway can promote the phosphorylation of 4E-BP1 and inhibit the activity of ribosomal protein S6 kinase (s6k) and 4E-BP1, ultimately leading to enhanced protein synthesis (Seiliez et al., 2008). In this experiment, dietary supplementation with Hyp significantly up-regulated the expression of *tor*, and *igf-1*, with *tor* showing the highest expression in the LF2 group and *igf-1* in the LF4 group in muscle. In the hepatopancreas, the LF4 group exhibited the highest expression levels of *both tor* and *igf-1*. Also, Western blot results showed that dietary Hyp supplementation significantly up-regulated the protein expression of P-4E-BP1 and P-4E-BP1/4E-BP1. This finding is consistent with the finding of the triploid crucian carp study (Cao et al., 2022). The PI3K/AKT pathway positively regulates the TOR pathway, and AKT phosphorylation can activate the TOR pathway (Mu et al., 2023; Yan et al., 2021). The data from this experiment showed that addition of Hyp in the dietary significantly up-regulated the protein expression of AKT, P-AKT and P-AKT/AKT. The above results illustrate that supplementation of Hyp in low fishmeal promotes collagen synthesis and improves muscle quality.

The taste of muscle is not only influenced by its flavor but also by the characteristics and type of muscle fiber. Hypertrophy of myofiber defined by an augment in the diameter or size of the myofiber and myofiber hyperplasia defined by an augment in the number of myofibers are two processes associated with myofiber growth (White et al., 2018). In general, an increase in myofiber density and a decrease in myofiber diameter are among the reasons that lead to an increase in muscle chewiness, thereby improving muscle quality (Dunajski, 1980; Espe, 2008; Johnston et al., 2000; Lin et al., 2009). In simpler terms, finer myofibers contribute to the production of high-quality muscle, while the opposite is true for low-quality muscle (Lin et al., 2009). A significant increase in the density of myofibers and a reduction in the diameter of myofibers was observed in this experiment with Hyp supplementation. This corresponds to the improvement of muscle texture with Hyp. The type of fiber of the muscle can also determine muscle quality, which can influence in terms of flavor and texture (Lefaucheur, 2010). Shrimp have two types of myofibers, fast and slow. Fast myofibers contain a lot of ATPase, which can cause a rapid decline in pH levels in the muscle after the animal dies (Immonen et al., 2000). Thus, prompting a shift from fast to slow myofibers can improve muscle quality. In the results of this experiment in which the addition of Hyp down-regulated the expression of mRNA in fast myofibers (*smyhc1*, *smyhc 2*, and *smyhc6a*), while the opposite was true for mRNA in slow myofibers (*smyhc 5*, *smyhc 15*). The type of myofiber can affect the sarcomere diameter. Slow muscle fibers have longer sarcomeres, which can form a protective layer over the muscle. This layer of structure helps to prevent the degradation of soluble proteins, which can improve the overall quality of the muscle (Ertbjerg and Puolanne, 2017; Wu et al., 2018). In the TEM results it could be observed that the sarcomere diameter of the shrimp fed with Hyp supplementation was significantly longer than that of the LF0 group. This is consistent with the up-regulation of slow muscle fiber expression after dietary hydroxyproline supplementation. Therefore, Hyp may be able to change muscle fiber types and characteristics thus improving flesh strength.

In conclusion, the results of this experimental study indicate that the addition of Hyp had a positive effect on the growth of *L. vannamei*. The supplementation of 0.4% Hyp in the dietary improved the muscle quality of *L. vannamei*, improved taste evaluation, and increased the collagen content thus improving the nutritional value of the muscle.

## Author contributions

**Menglin Shi:** Conceptualization, Formal analysis, Investigation, Writing-original draft. **Xiaohui Dong**, **Shuyan Chi**, **Qihui Yang**, **Hongyu Liu**, **Junming Deng**, **Shuang Zhang**, **Beiping Tan:** Methodology, Resources. **Haoming** **Li**, **Tianyu Chen**, **Bocheng Huang**, **Xiaoyue Li:** Methodology, Resources. **Shiwei Xie:** Methodology, Supervision, Writing-review & editing, Project administration, Funding acquisition.

## Declaration of competing interest

We declare that we have no financial and personal relationships with other people or organizations that can inappropriately influence our work, and there is no professional or other personal interest of any nature or kind in any product, service and/or company that could be construed as influencing the content of this paper.
