# Supplementary material for: Effects of hydroxyproline supplementation in low fish meal diets on collagen synthesis, myofiber development and muscular texture of juvenile Pacific white shrimp (Litopenaeus vannamei)
Source: Anim Nutr. 2024 Mar 28;17:428–37. doi: 10.1016/j.aninu.2024.01.013 (PMC11163151; doi:10.1016/j.aninu.2024.01.013)
Supplement: Multimedia component 1 [file mmc1.docx]

**Table S1** Formulation and proximate of experimental diets (% dry matter).

| Ingredients | Diets | | | | | |
| --- | --- | --- | --- | --- | --- | --- |
|  | HF | LF0 | LF2 | LF4 | LF6 | LF8 |
| Fish meal ^1^ | 25.00 | 10.00 | 10.00 | 10.00 | 10.00 | 10.00 |
| Soybean meal^2^ | 23.00 | 23.00 | 23.00 | 23.00 | 23.00 | 23.00 |
| Peanut meal ^3^ | 10.00 | 10.00 | 10.00 | 10.00 | 10.00 | 10.00 |
| Shrimp shell meal ^4^ | 5.00 | 5.00 | 5.00 | 5.00 | 5.00 | 5.00 |
| Beer yeast ^5^ | 3.00 | 3.00 | 3.00 | 3.00 | 3.00 | 3.00 |
| Wheat flour ^6^ | 23.45 | 23.45 | 23.45 | 23.45 | 23.45 | 23.45 |
| *Clostridium autoethanogenum* protein^7^ | 0.00 | 7.25 | 7.25 | 7.25 | 7.25 | 7.25 |
| Soy protein concentrate ^8^ | 0.00 | 7.25 | 7.25 | 7.25 | 7.25 | 7.25 |
| Fish oil | 1.35 | 2.70 | 2.70 | 2.70 | 2.70 | 2.70 |
| Soybean oil | 2.50 | 2.50 | 2.50 | 2.50 | 2.50 | 2.50 |
| Soybean lecithin | 1.00 | 1.00 | 1.00 | 1.00 | 1.00 | 1.00 |
| Hydroxyproline ^9^ | 0.00 | 0.00 | 0.20 | 0.40 | 0.60 | 0.8 |
| Vitamin C | 0.10 | 0.10 | 0.10 | 0.10 | 0.10 | 0.10 |
| Choline chloride | 0.30 | 0.30 | 0.30 | 0.30 | 0.30 | 0.30 |
| CaH_2_PO₄ | 1.00 | 2.50 | 2.50 | 2.50 | 2.50 | 2.50 |
| Vitamin and mineral premix ^10^ | 1.00 | 1.00 | 1.00 | 1.00 | 1.00 | 1.00 |
| Cellulose microcrystalline | 3.25 | 0.8 | 0.6 | 0.4 | 0.2 | 0.00 |
| Methionine | 0.00 | 0.10 | 0.10 | 0.10 | 0.10 | 0.10 |
| Ethoxyquin | 0.05 | 0.05 | 0.05 | 0.05 | 0.05 | 0.05 |
| Total | 100.00 | 100.00 | 100.00 | 100.00 | 100.00 | 100.00 |
| Proximate composition |  |  |  |  |  |  |
| Dry matter | 90.50 | 90.33 | 91.00 | 90.00 | 91.33 | 89.95 |
| Crude protein | 39.16 | 38.89 | 38.85 | 39.98 | 38.55 | 38.38 |
| Crude lipid | 8.31 | 9.00 | 9.00 | 8.25 | 8.91 | 9.00 |

^1^ Fishmeal: Peruvian fishmeal, 71.85% crude protein, 9.30% crude lipids, provided by Technology de Alimentos S.A., Callao, Peru.

^2^ Soybean meal: 51.82% crude protein, 1.16% crude lipids, provided by Zhanjiang Hengxing aquatic Technology Co. Ltd., Zhanjiang, China.

^3^ Peanut meal: 58.91% crude protein, 1.5% crude lipids, provided by Zhanjiang Hengxing aquatic Technology Co. Ltd., Zhanjiang, China.

^4^ Shrimp shell meal: 39.27% crude protein, 0.49% crude lipids, provided by Zhanjiang Hengxing aquatic Technology Co. Ltd., Zhanjiang, China.

^5^ Beer yeast: 47.722% crude protein, 0.42% crude lipids, provided by Zhanjiang Hengxing aquatic Technology Co. Ltd., Zhanjiang, China.

^6^ Wheat flour: 12.26% crude protein,0.42% crude lipids, provided by Zhanjiang Hengxing aquatic Technology Co. Ltd., Zhanjiang, China.

^7^ *Clostridium autoethanogenum* protein (CAP): 84.20% crude protein, 0.19% crude lipids, provided by Hebei Shoulang New Energy Technology Co. Ltd., Tangshan, China.

^8^ Soy protein concentrate: 69.53% crude protein, 1.00% crude lipids, provided by Zhanjiang Hengxing aquatic Technology Co. Ltd., Zhanjiang, China.

^9^ Hydroxyproline was supplied by Shanghai Aladdin Biochemical Technology Co., Ltd., Shanghai, China.

^10^ Vitamin and mineral premix includes the following contents per kilogram of diet: thiamine, 5 mg; riboflavin, 10 mg; vitamin A, 5000 IU; vitamin D3, 1000 IU; vitamin E, 40 mg; menadione, 10 mg; pyridoxine, 10 mg; biotin, 0.1 mg; cyanocobalamin, 0.02 mg; calcium pantothenate, 20 mg; folic acid, 1 mg; niacin, 40 mg; vitamin C, 150 mg; FeSO_4_·H_2_O, 303 mg; KIO_3_, 1.3 mg; Cu_2_(OH)_3_Cl, 5 mg; ZnSO_4_·H_2_O, 138 mg; MnSO_4_·H_2_O, 36 mg; Na_2_SeO_3_, 0.6 mg; CoCl_2_·6H_2_O, 0.8 mg. The premix was provided by Beijing Enhalor International Tech Co., Ltd., Beijing, China.

**Table S2** Amino acid composition of the diets (% dry matter).

| Item Diets^1^ | | | | | |  |  |  |
| --- | --- | --- | --- | --- | --- | --- | --- | --- |
|  | HF | LF0 | LF2 | LF4 | LF6 | LF8 | CAP | SCP |
| Aspartic acid | 3.68 | 3.97 | 4.05 | 4.04 | 4.03 | 3.76 | 9.54 | 7.92 |
| Threonine | 1.43 | 1.48 | 1.51 | 1.51 | 1.50 | 1.43 | 4.02 | 2.86 |
| Serine | 1.70 | 1.75 | 1.77 | 1.78 | 1.76 | 1.70 | 3.21 | 3.59 |
| Glutamic acid | 6.39 | 6.50 | 6.63 | 6.60 | 6.58 | 6.45 | 9.78 | 12.82 |
| Glycine | 1.93 | 1.79 | 1.82 | 1.87 | 1.86 | 1.84 | 3.87 | 2.88 |
| Alanine | 1.91 | 1.96 | 1.87 | 1.87 | 1.86 | 1.84 | 4.63 | 2.97 |
| Cystine | 0.45 | 0.49 | 0.52 | 0.52 | 0.51 | 0.48 | 0.71 | 0.75 |
| Valine | 1.70 | 1.80 | 1.85 | 1.84 | 1.84 | 1.81 | 5.44 | 3.25 |
| Methionine | 0.76 | 0.79 | 0.81 | 0.80 | 0.80 | 0.74 | 2.29 | 0.68 |
| Isoleucine | 1.52 | 1.70 | 1.74 | 1.73 | 1.74 | 1.71 | 5.28 | 3.19 |
| Leucine | 2.71 | 2.78 | 2.85 | 2.83 | 2.82 | 2.81 | 6.38 | 5.43 |
| Phenylalanine | 1.72 | 1.79 | 1.83 | 1.81 | 1.81 | 1.82 | 3.30 | 3.68 |
| Lysine | 2.24 | 2.32 | 2.37 | 2.38 | 2.37 | 2.34 | 8.70 | 4.34 |
| Histidine | 0.94 | 0.88 | 0.88 | 0.88 | 0.89 | 0.89 | 1.68 | 1.79 |
| Arginine | 2.52 | 2.53 | 2.53 | 2.57 | 2.60 | 2.53 | 3.40 | 5.21 |
| Proline | 1.97 | 1.97 | 1.98 | 2.03 | 2.02 | 1.97 | 2.40 | 3.55 |
| Hydroxyproline | 0.19 | 0.11 | 0.33 | 0.47 | 0.62 | 0.78 | 0 | 0.17 |
| Total | 33.76 | 34.61 | 35.34 | 35.53 | 35.61 | 34.90 | 74.63 | 65.08 |

CAP = *Clostridium autoethanogenum* protein; SCP = soy protein concentrate.

^1^HF = high fishmeal (25% fishmeal content); LF0 = low fishmeal (10% fishmeal content); LF2 = LF0 + 0.2 g/kg hydroxyproline; LF4 = LF0 + 0.4 g/kg hydroxyproline; LF6 = LF0 + 0.6 g/kg hydroxyproline; LF8 = LF0 + 0.8 g/kg hydroxyproline.

**Table S3** Primers used for quantitative real-time PCR.

| Gene | Forward primers (5′ − 3′) | Reverse primers (5′ − 3′) | Accession no. |
| --- | --- | --- | --- |
| *tgf-β* | CCATTCACTGTCCAGCTATGT | CTTTCAGCAGGGACACTCTATC | >XM_027378574.1 |
| *igf-1* | TTCGTGATTCTGGGACAAGG | CCCAGGAATCAGTACCATCATC | >XM_027365614.1 |
| *tor* | TGTAGCTGGTGGAGCATTTAG | CCCATGTCTTTGCGCTTTG | >XM_027381629.1 |
| *smych1* | CGGTGCCTCTGAGAAGAAAG | AGGAGTTGTCGTTACGGGTG | >XM_027371418.1 |
| *smych 2* | CGATATTTACGACTACCGCTACG | CGACCCCTCTGCTTGAACTT | >XM_027368558.1 |
| *smych 6a* | ATCCGAACTTGCTGATGCC | TCAGCACGGAGTTCGTCAGC | >XM_027368559.1 |
| *smych 5* | ATGCTCAACGAAGCCAGACA | CCTTCATCGCATTTGTTTCG | >XM_027359018.1 |
| *smych 15* | GCAACTACGCCACCGAACAC | CCTCACCGATCTGGTCCATCAA | >XM_027352482.1 |
| *col1a1* | CTGGAAGCCTGTTCGGAATAA | CACATCGTTGGTGAAGAGGTAG | >XM_027350742.1 |
| *col1a2* | GCCAGGATTCCAGGGATTAG | GCCTACCAACATCTCCCTTT | >XM_027369130.1 |
| *myo15* | CGGACGTGGAACAAGTATGA | TTGATGTACCGGTTGGACAC | >XM_027369062.1 |
| Beta-actin | GAGCAACACGGAGTTCGTTGT | CATCACCAACTGGGACGACATGGA | AF300705.2 |

*tgf-β* *=* transforming growth factor-beta receptor-associated protein 1-like; *igf-1 =* insulin-like growth factor-binding protein-related protein 1; *tor =* target of rapamycin complex 2 subunit mapkap1 -like; *smych* *1*, *smych* *2*, *smych* *6a*, *smych* *5*, *smych* *15 =* myosin heavy chain gene family; *col1a1 =* collagen alpha-1(I) chain-like; *col1a2 =* collagen alpha-2(I) chain-like; *myo15 =* collagen alpha-2(XI) chain-like.

**Table S4** Muscle composition of *L. vannamei* fed different diets.

| Index | Diet^1^ |  |  |  |  |  |
| --- | --- | --- | --- | --- | --- | --- |
|  | HF | LF0 | LF2 | LF4 | LF6 | LF8 |
| Moisture, % | 75.33±0.333 | 74.67±0.882 | 75.00±0.577 | 75.33±0.333 | 75.0±1.000 | 76.33±0.333 |
| Crude lipid, % | 0.82±0.060 | 0.92±0.067 | 1.07±0.033 | 0.82±0.076 | 0.95±0.029 | 0.80±0.058 |
| Crude protein, % | 22.28±0.256 | 22.23±0.373 | 22.67±0.113 | 22.77±0.188 | 22.98±0.151 | 21.96±0.302 |

^1^HF = high fishmeal (25% fishmeal content); LF0 = low fishmeal (10% fishmeal content); LF2 = LF0 + 0.2 g/kg hydroxyproline; LF4 = LF0 + 0.4 g/kg hydroxyproline; LF6 = LF0 + 0.6 g/kg hydroxyproline; LF8 = LF0 + 0.8 g/kg hydroxyproline.
